# Evidence-based nutrition for the very preterm infant in 2024^☆^

**DOI:** 10.1016/j.jped.2024.08.002

**Published:** 2024-08-30

**Authors:** Sarah N. Taylor

**Affiliations:** Yale University School of Medicine, New Haven, CT, USA

Mother's milk is the gold standard nutrition for the full-term newborn.^1^ For the preterm newborn, the situation is more complicated. First, depending on how early the preterm infant is born, the infant misses a portion of nutrients delivered at high concentrations at the end of pregnancy such as calcium, phosphorus, and fatty acids. Secondly, human milk protein content is lower than the amount of protein delivered by the placenta. Thirdly, the hospitalized preterm infant may have higher protein and energy needs than either the fetus or the full-term, healthy newborn infant. Fourthly, although human milk may be lacking in nutrition to preterm infants, it is an important protector in its similarities to amniotic fluid with important growth factors for the development of the gut and other organs. Fifthly, human milk also delivers a large bolus of immunoregulatory factors to the preterm infant who is at risk for both infectious and inflammatory diseases such as necrotizing enterocolitis.^2^ Therefore, the optimal nutrition for a preterm infant may be human milk intake with the addition of those nutrients delivered in lower concentrations in milk compared to the placenta.

The term “may be” in the preceding sentence is purposeful because, even when an approach to nutrition is designed to match fetal and early life needs, it must have clinical evidence of a positive impact on infant outcomes to be considered the gold standard. An outcome commonly studied in preterm infant nutrition studies is infant growth. In this issue, Soldateli and colleagues^3^ compare how nutritional intake, described as three types of very preterm infant feeding, relates to the outcomes of infant weight and head circumference growth during birth hospitalization. For this study, very preterm infant outcomes included weight and head circumference at hospital discharge and the trajectory of weight and head circumference growth throughout the hospitalization. This study includes data from the Vermont Oxford Network on 4062 surviving very preterm infants from 12 Brazilian Neonatal Intensive Care Units (NICUs). To determine how this study's results impact the field, it is important to discuss the data with which this study was performed, how the feeding type at discharge characterizes nutritional practices in Brazilian NICUs, and what the study's growth outcomes represent for very preterm infants.

The Vermont Oxford Network database utilized in this study has a track record of providing data on nutritional practice and in-hospital growth for very preterm infants.^4^^,^^5^ The benefit of this database for research is that it includes multi-hospital, existing data which is collected with specific data definitions. The shortcomings of this database for research are the limitations in collected data with growth measured only at birth and hospital discharge and feeding type measured only at discharge. Only assumptions can be made about what happened for nutrition and growth in between the two time points. Therefore, this cross-sectional study serves as a resource for building clinical approaches and further research studies rather than as a definitive statement on the nutrition and growth of very preterm infants.

The extent of growth faltering in hospitalized very preterm infants was made startlingly evident twenty-five years ago by Ehrenkranz et al.^6^ and has been detailed in further research such as this study. Although growth faltering, defined as not achieving expected growth patterns, is an undesirable outcome, the importance of this result is magnified because growth patterns relate to neurodevelopmental outcomes. In most studies, higher gains in growth relate to higher scores on neurodevelopmental assessments.^7^^,^^8^ Yet, as the relationship between growth patterns and neurodevelopment is examined in more detail, great complexity is evident. For example, it is not known what specific macronutrient, micronutrient, vitamin, or combination of nutrition are responsible for why growth relates positively to neurodevelopment.^8^ It also is not known how genetic potential, prenatal factors, social determinants of health, NICU stress, and health morbidities impact this association between growth and neurodevelopment.^8^, ^9^, ^10^ Lastly, the specific growth trajectory that predicts higher neurodevelopmental assessment scores has not been identified.^7^, ^8^, ^9^, ^10^ Therefore, the current state of this science is that less growth faltering is preferred, but the specific growth patterns that predict optimal outcomes remain unknown.

In the Soldateli and colleagues,^3^ article, three feeding types are compared which are exclusive human milk (no formula or fortifier), mixed diet (human milk with fortifier or formula), and exclusive formula. The growth of infants receiving each feeding type is standardized to the Fenton intrauterine-based growth curve and then compared between groups. All three groups exhibited weight and head circumference z-score averages at least one standard deviation (−1) below the mean of the Fenton growth curve. Although the infants fed human milk and mixed diets were “lighter and had smaller heads at discharge/transfer”, the formula-fed infants were also light and had small heads. The conclusion from this study comparing feeding types is that nutritional inadequacies were highest for the infants fed exclusively human milk followed by a mixed diet followed by exclusive formula at hospital discharge. Infant formula, received by the exclusive formula group and the mixed diet group, and human milk fortifier, received by the mixed diet group, deliver greater concentrations of some nutrients than exclusive human milk. Feeding products such as preterm infant formula and human milk fortifier are formulated to provide the nutrients that are delivered at high concentrations in the third trimester of pregnancy such as calcium, phosphorus, and fatty acids, and the protein content to match fetal accretion in the third trimester.^11^

Just as with growth patterns, in theory, preterm infant nutrition should match fetal and early-life nutritional needs. Also, how nutritional support relates to very preterm infant outcomes warrants consideration. In the study of how infant formula and human milk fortifier intake impact very preterm infant outcomes, meta-analysis and systematic reviews of randomized controlled trials were performed which compare infant formula to donor milk and fortified human milk with unfortified human milk. In these studies, infants fed either preterm infant formula or human milk fortifier diets exhibit higher growth gains but with no difference in neurodevelopment.^12^^,^^13^

One macronutrient studied specifically for how it relates to both growth and neurodevelopment is protein. In the Soldateli and colleagues,^3^ article, although the specific types of nutrition are not detailed, it is likely that the three feeding groups differed in protein intake with exclusive formula feeding as the highest and exclusive human milk feeding as the lowest. Current evidence points to a protein intake between 3.9 and 4.5 g/kg/day to promote short-term growth in very preterm infants.^14^ For exclusive human milk feeding to achieve 3.9–4.5 g/kg/day protein intake, the infant must feed 180–450 mL/kg/day depending on the protein concentration of the milk. Therefore, most very preterm infants receiving exclusive human milk do not receive the protein intake associated with optimal growth. Less is known as to how protein intake directly impacts neurodevelopment, but the current assumption is that at least a portion of the association between growth patterns and neurodevelopment relates to protein intake.^8^

Based on the Soldateli and colleagues,^3^ article, what are some considerations for the optimization of growth in Brazilian NICUs? The first is to consider opportunities for greater attention to infant growth. The lack of change in growth patterns over the nine-year study period may reflect a sustained inadequacy in attention to growth in Brazilian NICUs at a time when NICUs elsewhere in the world have incorporated regular assessments of infant growth adequacy into daily NICU rounds. Regular assessment of growth relates to higher growth rates.^15^

The second consideration is to protect the excellent rates of human milk intake in Brazilian NICUs. As outlined by Soldateli and colleagues, in the United States, NICUs have increased very preterm infant growth parameters and intake of mother's milk, yet mother's milk feedings in the United States remain far below those in Brazilian NICUs.^5^ The rate of 84% of very preterm infants receiving some amount of human milk at discharge in the NICUs in the Soldateli et al. study must be sustained as Brazilian NICUs consider methods to improve very preterm infant growth. Intake of mother's milk relates positively to very preterm infant neurodevelopment, and it is not yet known whether the intake of mother's milk is more important than the impact of nutrition on long-term very preterm infant outcomes.^16^ Additionally, this rate of human milk feedings may benefit short-term outcomes such as the low rate of necrotizing enterocolitis in the Soldateli et al.’s^3^ study. Of note, donor milk does not appear to have the same association with neurodevelopment as mother's milk. In randomized, controlled trials, neurodevelopmental outcomes for donor milk- and formula-fed infants were similar.^12^^,^^17^

The third consideration is to increase nutrient delivery to human milk-fed very preterm infants in Brazilian NICUs. The most straightforward way to do this is to standardize the addition of human milk fortifier to human milk feeds for hospitalized very preterm infants. As mentioned, in the meta-analysis of randomized, controlled trials, preterm infants fed human milk fortifier have higher growth gains than those fed unfortified human milk. For most of these studies, the human milk fortifier was a multi-component human milk fortifier that includes not only protein but also other nutrients important in the third trimester such as calcium, phosphorus, and fatty acids. However, it is also important to recognize that, although multi-component human milk fortifiers have been related to better growth, it has not been related to better neurodevelopment scores.^13^ This suggests that the relationship between infant growth patterns and neurodevelopment is more complex than the delivery of nutrients.

The fourth consideration is to recognize that the optimal nutrition for very preterm infants is an evolving field of study. I titled this editorial “Evidence-based Nutrition for the Very Preterm Infant in 2024″ because the recommendations in this article are only as strong as the state of the science in 2024. Research is ongoing to identify the best growth metrics, the best nutritional intake, and how both relate to genetic potential, maternal and infant disease exposures, NICU stress, and social determinants of health. The article by Soldateli et al.^3^ reflects the potential for improved growth monitoring and nutrition delivery in Brazilian NICUs, but further research is needed before an optimal very preterm infant growth pattern is determined or the “gold standard” very preterm infant nutrition is identified.

## Conflicts of interest

The authors declare no conflicts of interest.
